# Memory Trace in Feeding Neural Circuitry Underlying Conditioned Taste Aversion in *Lymnaea*

**DOI:** 10.1371/journal.pone.0043151

**Published:** 2012-08-10

**Authors:** Etsuro Ito, Emi Otsuka, Noriyuki Hama, Hitoshi Aonuma, Ryuichi Okada, Dai Hatakeyama, Yutaka Fujito, Suguru Kobayashi

**Affiliations:** 1 Laboratory of Functional Biology, Kagawa School of Pharmaceutical Sciences, Tokushima Bunri University, Sanuki, Japan; 2 Division of Biological Sciences, Graduate School of Science, Hokkaido University, Sapporo, Japan; 3 Complex Systems Research Group, Research Institute for Electronic Science, Hokkaido University, Sapporo, Japan; 4 Department of Systems Neuroscience, School of Medicine, Sapporo Medical University, Sapporo, Japan; Tokai University, Japan

## Abstract

**Background:**

The pond snail *Lymnaea stagnalis* can maintain a conditioned taste aversion (CTA) as a long-term memory. Previous studies have shown that the inhibitory postsynaptic potential (IPSP) evoked in the neuron 1 medial (N1M) cell by activation of the cerebral giant cell (CGC) in taste aversion-trained snails was larger and lasted longer than that in control snails. The N1M cell is one of the interneurons in the feeding central pattern generator (CPG), and the CGC is a key regulatory neuron for the feeding CPG.

**Methodology/Principle Findings:**

Previous studies have suggested that the neural circuit between the CGC and the N1M cell consists of two synaptic connections: (1) the excitatory connection from the CGC to the neuron 3 tonic (N3t) cell and (2) the inhibitory connection from the N3t cell to the N1M cell. However, because the N3t cell is too small to access consistently by electrophysiological methods, in the present study the synaptic inputs from the CGC to the N3t cell and those from the N3t cell to the N1M cell were monitored as the monosynaptic excitatory postsynaptic potential (EPSP) recorded in the large B1 and B3 motor neurons, respectively. The evoked monosynaptic EPSPs of the B1 motor neurons in the brains isolated from the taste aversion-trained snails were identical to those in the control snails, whereas the spontaneous monosynaptic EPSPs of the B3 motor neurons were significantly enlarged.

**Conclusion/Significance:**

These results suggest that, after taste aversion training, the monosynaptic inputs from the N3t cell to the following neurons including the N1M cell are specifically facilitated. That is, one of the memory traces for taste aversion remains as an increase in neurotransmitter released from the N3t cell. We thus conclude that the N3t cell suppresses the N1M cell in the feeding CPG, in response to the conditioned stimulus in *Lymnaea* CTA.

## Introduction

The pond snail *Lymnaea stagnalis* is capable of acquiring different forms of associative learning, including both classical and operant conditioning. In addition to acquisition of learning, *Lymnaea* individuals are able to consolidate the learning into long-term memory (LTM) [1]–[4]. One remarkable learning ability in *Lymnaea* is the capacity to establish taste aversion and consolidate it into LTM. This is referred to as conditioned taste aversion (CTA) [5]–[7]. To produce a CTA in *Lymnaea*, an appetitive stimulus (e.g., sucrose) is used as the conditioned stimulus (CS). Application of the CS to the lips increases the feeding response in snails. An aversive stimulus (e.g., KCl) is used as the unconditioned stimulus (US). Application of the US to the snails inhibits feeding behavior. In the taste aversion-training procedure, the CS is paired with the US. After repeated temporal contingent presentations of the CS and US, the CS no longer elicits a feeding response, and this taste aversion persists for more than a month [8].

On the basis of these behavioral experiments, we proposed a neuromodulatory model for *Lymnaea* CTA in which the association of the CS and US resulting from the taste aversion training potentiates an inhibitory polysynaptic pathway, resulting in a suppression of the feeding response to the CS [9], [10]. From knowledge of the underlying neural circuit [11]–[13], we found that the chemosensory inputs resulting from the CS and US were associated in the cerebral giant cells (CGCs) [14], [15], and that the polysynaptic inhibitory influence of the CGCs on the neuron 1 medial (N1M) cells was enhanced during the CTA period [9], [10]. Because the N1M cells are part of the central pattern generator (CPG) for feeding, together with a number of other interneurons [11], [12], [16], the enhancement of the inhibitory influence is thought to suppress the feeding response in *Lymnaea* CTA.

On the other hand, Benjamin and his colleagues have so far analyzed the mechanism underlying taste appetitive training (i.e., reward conditioning) by use of the same *Lymnaea* feeding system [17]. As expected, they demonstrated that the taste appetitive training activated the feeding CPG [18]. Further, they examined the interaction between the two feeding CPG interneurons, which were the neuron 3 tonic (N3t) and N1M cells. They demonstrated that there was a reduction in the number of N3t cell spiking at some hours after the training procedure, and this reduction was correlated with an increase in the conditioned fictive feeding response. Taking into account that the N3t cell tonically inhibits the N1M cell by monosynaptic connection [19], their computer simulation data for the N3t-N1M interaction suggested that a change in the N3t firing was sufficient to explain an increase in the fictive feeding activity produced by taste appetitive training [20].

**Figure 1:**
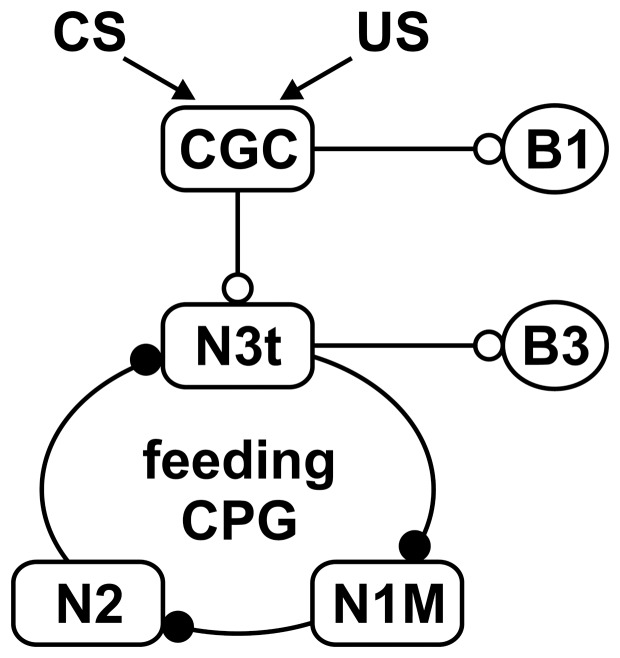
Schematic presentation of the neural circuitry underlying taste aversion training in the *Lymnaea* CNS. The signals of the CS and US are associated in the CGC. Rectangles and circles indicate interneurons and motor neurons, respectively. At synapses, open circles and closed circles indicate excitatory monosynaptic inputs and inhibitory monosynaptic inputs, respectively. The N1M, N2 and N3t cells form part of the feeding CPG. The EPSPs recorded in the B1 and B3 motor neurons can be used for monitoring the changes in the synaptic connections of CGC-N3t and N3t-N1M, respectively.

From the results by Benjamin and his colleagues [20], we hypothesized that our taste aversion training would result in (1) enhancement of the excitatory synaptic connection from the CGC to the N3t cell and (2) enhancement of the inhibitory synaptic connection from the N3t cell to the N1M cell (Figure 1). However, because the N3t cell is too small to access consistently by electrophysiological methods, the excitatory monosynaptic responses of the N3t cell that were induced by activation of the CGC and the inhibitory monosynaptic inputs from the N3t cell to the N1M cell were monitored as the monosynaptic excitatory postsynaptic potentials (EPSPs) recorded in the large B1 and B3 motor neurons, respectively. The B1 motor neuron receives the monosynaptic EPSP from the CGC [21], and thus the monitoring of the B1 EPSPs that are induced by activation of the CGC corresponds to the N3t EPSPs. The size of spontaneous EPSP of the B3 motor neuron corresponds to the amount of neurotransmitter released from the N3t cell [19], [22], [23].

In the present study, we first examined a change in the amplitude of monosynaptic EPSPs of the B1 motor neuron that was induced by activation of the CGC in the central nervous system (CNS) isolated from the taste aversion-trained snails. We then examined the spontaneous monosynaptic EPSPs of the B3 motor neuron in the same CNS. Our results lead us to conclude that an increase in neurotransmitter released from the N3t cell to the N1M cell in the circuit of CGC-N3t-N1M cells determines the behavioral change by the taste aversion training, as well as that by the taste appetitive training.

## Methods

### Snails

Specimens of *Lymnaea stagnalis* (L.) with a 18–23 mm shell were obtained from our snail-rearing facility (original stocks from Vrije Universiteit Amsterdam). All snails were maintained in dechlorinated tap water (i.e., pond water) under a 12∶12 light-dark cycle at 20°C and fed *ad libitum* on a kind of rape *Brassica rapa var. peruviridis* (*Komatsuna* [in Japanese]) and a spiral shell food (Nisso, Saitama, Japan) every other day. *Lymnaea* exhibit good growth and reproduction under these feeding conditions.

### Paradigm for taste aversion training

All snails were first given a pretest in polystyrene petri dishes (diameter 35 mm) [24], [25]. In this observation period (1 min), the number of feeding responses (rasping movements of the buccal mass) was counted in distilled water following a 15-sec application of 10 mM sucrose (the CS) to the lips of the snail. Taste aversion training was brought about by pairing the 10 mM sucrose CS and the 10 mM KCl US. The duration of both the CS and the US was 15 sec, with an interstimulus interval between the onsets of the CS and US of 15 sec. A 10-min intertrial interval was interposed between each pairing of the CS-US. Snails received 10 paired CS-US trails. We also used a backward-conditioned (US-CS) control group and a naive control group to validate associative learning. For the naive control group, only distilled water was applied to the lips instead of the CS and US. In the posttest sessions at 10 min after the end of training, snails were again challenged with the CS, and the number of bites was recorded over a 1-min interval in distilled water after a 15-sec application of the CS. All tests were performed blindly. The behavioral experiments were performed in the morning, because the learning scores are better in the morning than at other times [26].

### Electrophysiology

Three to six hours after the training, the CNS was isolated from snails in *Lymnaea* saline (50 mM NaCl, 1.6 mM KCl, 2 mM MgCl_2_, 3.5 mM CaCl_2_, 10 mM Hepes, pH 7.9 adjusted by NaOH) [27]. The snails used for electrophysiology were randomly selected from among those subjected to training. The isolated CNS was digested with protease (proteinase type XXIV, Sigma-Aldrich, St. Louis, MO, USA) dissolved at 1 mg/ml in the *Lymnaea* saline for 10 min at room temperature. The membrane potentials of the CGC and the B1 motor neurons were simultaneously recorded in HiDi/Hex saline (35 mM NaCl, 2 mM KCl, 14 mM MgCl_2_, 8 mM CaCl_2_, 10 mM HEPES, 1 mM hexamethonium chloride, pH 7.9 adjusted by NaOH) [27]. This HiDi/Hex saline was used to isolate the monosynaptic excitatory CGC-B1 synapse from, if any, polysynaptic CGC-B1 interactions. As described in the Introduction, the synaptic transmission from the CGC to the B1 motor neuron is thought to be monosynaptic [21]. But because we recorded compound EPSPs (see below), we needed to ensure the monosynaptic responses by use of the HiDi/Hex saline.

The cells were impaled with glass microelectrodes filled with 2 M potassium acetate. The resistance of these microelectrodes ranged from 50 to 70 MΩ. We intracellularly recorded a compound EPSP in the B1 motor neuron that was evoked by bursting action potentials in the CGC [28]. The constant depolarizing current was injected into the CGC for 1 s to elicit the bursting action potentials using the amplifier's built-in function for current injection. The single EPSP in the B1 motor neuron is so small that we monitored the compound EPSP for an activation period of 1 s. We adjusted the magnitude of the depolarizing current to elicit 10 spikes in the CGC during a 1-s current injection. However, the adjustment for 10 spikes/s in the CGCs was difficult in some cases. Thus, we collected the data presenting the 8–11 spikes in the CGCs during a 1-s current injection. The EPSPs were recorded 5 times from a single B1 motor neuron. After the recording of the B1 monosynaptic EPSP, we changed the solution to normal *Lymnaea* saline, which was the same as that used for CNS dissection. Then, we impaled the B3 motor neurons with glass microelectrodes and recorded the spontaneous voltage responses. We analyzed the B1 EPSP with a noise level larger than 3.0 mV and the B3 EPSP with a noise level larger than 2.0 mV. The EPSPs were not recorded before training: they were recorded and compared among different training groups only after training.

### Statistics

The data are expressed as the mean ± SEM. The significant differences at the level of *P*<0.05 were examined by one-way ANOVA and posthoc Scheffé test.

## Results

### Learning score

**Figure 2:**
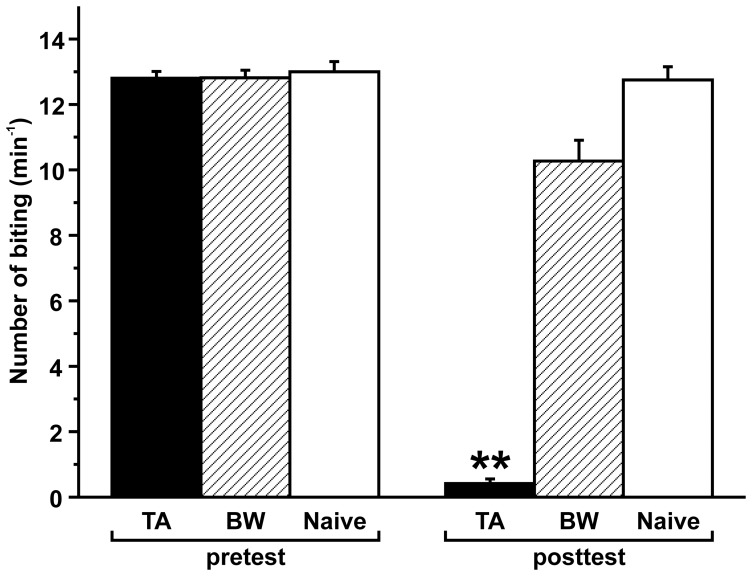
Learning scores by taste aversion training. After the CS was paired 10 times with the US in the taste aversion-training session, the feeding response to the CS was significantly reduced (**P<0.01) at the posttest of 10 min, compared to those observed for the backward-conditioned and naive control snails. In all cases in the pretest session, there were no significant differences (*P*>0.05) in the feeding response of the taste aversion-trained snails, backward-conditioned or naive control snails. TA, BW and naive indicate taste aversion-trained snails, backward-conditioned snails and naive control snails. The numbers of snails used were 55, 48 and 28 for the taste aversion-trained snails, backward-conditioned snails and naive control snails, respectively.

Snails were trained using the 10-trial training (i.e., CS – US pairing) procedure. We also prepared backward-conditioned snails (i.e., US – CS pairing) and naive snails as controls. In all groups of snails, a pretest to the CS was given and the number of bites/min was ascertained. At 10 min after the end of the taste aversion training, we found that the feeding response elicited by the CS (i.e., sucrose) in the posttest session was significantly reduced (*P*<0.01), compared to both the pretest session and to the posttest session of the backward-conditioned or the naive control group (Figure 2).

Moreover, in all cases, there were no significant differences (*P*>0.05) in the feeding response elicited in the pretest session of the taste aversion-trained snails, backward-conditioned snails or naive control snails.

### Evoked compound EPSPs recorded in the B1 motor neuron by activation of the CGC

Our previous studies have so far suggested that a change in the amount of neurotransmitter (i.e., serotonin) released from the CGCs by a cAMP/cAMP response element-binding cascade plays an important role at the synapses between the learning key neuron (i.e., CGC) and its following neurons in *Lymnaea* CTA [29]–[34]. In addition, the spikes in the CGC evoke one-to-one monosynaptic EPSPs in the N3t cell [12]. Therefore, it would be better if we could monitor a change in the EPSPs of the N3t cell by activation of the CGC in the taste aversion-trained snails. However, the N3t cells are small and it can be difficult to record their activity by intracellular electrophysiology in a reliable and consistent manner.

On the other hand, the B1 motor neuron is known to receive a monosynaptic excitatory input from the CGC [21]. We thus decided that it was more convenient to monitor a change in the B1 EPSPs instead of the N3t EPSPs by activation of the CGC in the taste aversion-trained snails. The CGC is serotonergic, and the B1 motor neuron uses two kinds of 5-HT receptors, the 5-HT_2_-like receptor and 5-HT_3_-like receptor [28], [29].

**Figure 3:**
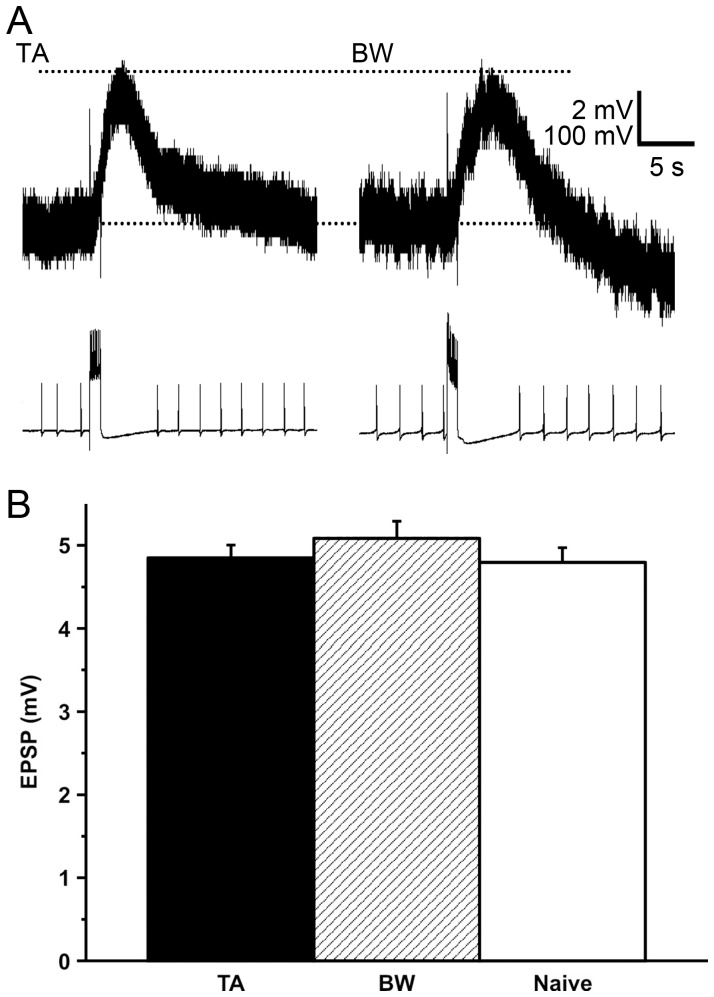
Evoked compound EPSPs in the B1 motor neuron by activation of the CGC. The data correspond to the strength of the CGC-N3t synaptic connection. **A.** Typical recordings of evoked compound B1 EPSPs. Left: a recording from a taste aversion-trained snail; right: a recording from a backward-conditioned snail. Upper: recordings of evoked compound B1 EPSPs; lower: recordings of spikes of the CGCs. We adjusted the magnitude of the depolarizing current to elicit about 10 spikes in the CGC during a 1-s current injection and monitored the compound EPSP evoked by this activation. **B.** There were no significant differences (*P*>0.05) in the B1 EPSPs of the taste aversion-trained snails, backward-conditioned and naive control snails. TA, BW and naive indicate taste aversion-trained snails, backward-conditioned snails and naive control snails. The numbers of EPSP recordings used were 74, 47 and 49. These data were accumulated at 3 to 6 h after the training, by 5 recordings for the isolated CNSs prepared from the 18 taste aversion-trained snails, 10 backward-conditioned snails and 13 naive control snails, respectively.

The amplitude of evoked compound EPSPs consisting of only monosynaptic CGC-B1 inputs, which was recorded in the CNSs isolated from the taste aversion-trained snails, was almost identical to that recorded in the CNSs isolated from the backward-conditioned and the naive control snails (*P*>0.05, Figure 3). The full width at half maximum of the EPSPs was also not significantly different (*P*>0.05) among the CNSs isolated from the following three groups: for taste aversion-trained snails, the value was 5.18±0.13 (s) from 74 recordings of 18 snails; for backward-conditioned snails, it was 5.65±0.16 (s) from 47 recordings of 10 snails; and for naive control snails, it was 5.48±0.15 (s) from 49 recordings of 13 snails. These data were recorded at 3–6 h after training. That is, our hypothesis (#1) that the excitatory monosynaptic connection from the CGC to the N3t cell was enhanced was rejected.

### Spontaneous single EPSP recorded in the B3 motor neuron

The B3 EPSPs were recorded because they act as monitors of N3t firing [19]. Spikes in the N3t cells generate one-to-one monosynaptic EPSPs on the B3 motor neurons [22], [23] and can therefore be used as an indirect method for recording N3t firing rates. Marra et al. used *Lymnaea* semi-intact preparations that included the whole CNS, lips and esophagus, and monitored the EPSPs evoked by the monosynaptic inputs from the N3t cell on the B3 motor neuron some hours after the taste appetitive training [20]. A reduction in the frequency of EPSP inputs was clearly observed after the taste appetitive training.

Based on the somewhat analogous study of Marra et al. [20], we expected to observe an increase in the frequency of B3 EPSPs after the taste aversion training. However, our experiments failed to meet this expectation. There were no significant differences among the following three groups (*P*>0.05): for taste aversion-trained snails, the value was 10.7±0.97 (EPSPs/9 s) for 7 snails; for backward-conditioned snails, it was 10.9±1.30 (EPSPs/9 s) for 9 snails; and for naive control snails, it was 14.7±1.66 (EPSPs/9 s) for 10 snails.

**Figure 4:**
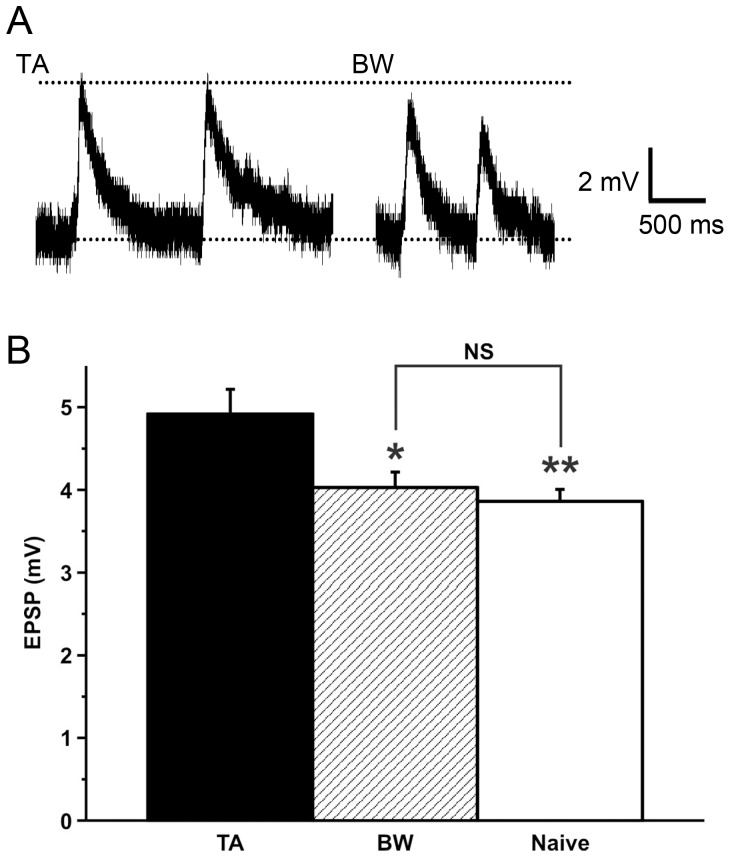
Spontaneous single EPSP in the B3 motor neuron. The data correspond to the strength of the N3t-N1M synaptic connection. **A.** Typical recordings of spontaneous B3 EPSPs. Left: a recording from a taste aversion-trained snail; right: a recording from a backward-conditioned snail. **B.** The EPSPs recorded in the B3 motor neurons from the taste aversion-trained snails were significantly larger (**P<0.01 and *P<0.05) than those observed for the backward-conditioned and naive control snails, respectively. There was no significant difference (*P*>0.05) in the EPSPs between the backward-conditioned and naive control snails. TA, BW and naive indicate taste aversion-trained snails, backward-conditioned snails and naive control snails. The numbers of EPSP recordings used were 64, 65 and 86. These data were accumulated at 3 to 6 h after the training for the isolated CNSs prepared from the 7 taste aversion-trained snails, 10 backward-conditioned snails and 10 naive control snails, respectively.

We then compared the amplitude of spontaneous single EPSPs that were recorded in the B3 motor neurons of the CNSs isolated from the taste aversion-trained snails, to that of the spontaneous single EPSPs recorded in the B3 motor neurons of the CNSs isolated from the backward-conditioned and the naive control snails (Figure 4). The B3 EPSP amplitude in the taste aversion-trained snails was significantly larger (*P<*0.01) than that in the backward-conditioned snails and also larger (*P<*0.05) than that in the naive control snails. There was no significant difference between those of the backward-conditioned and the naive control snails. These data were recorded at 3–6 h after training. That is, our hypothesis (#2) that the inhibitory synaptic connection from the N3t cell to the N1M cell was enhanced was strongly supported.

On the other hand, the full width at half maximum of the B3 EPSPs was also not significantly different (*P*>0.05) among the CNSs isolated from the three groups. For taste aversion-trained snails, the value was 0.17±0.02 (s) from 64 recordings of 7 snails; for backward-conditioned snails, it was 0.19±0.02 (s) from 65 recordings of 10 snails; and for naive control snails, it was 0.16±0.01 (s) from 86 recordings of 10 snails.

## Discussion

### Memory trace in the neural circuitry underlying *Lymnaea* CTA

We examined the two synaptic connections of the CGC-N3t-N1M cells by taste aversion-training procedure. We isolated monosynaptic responses in our experiments. During the training, the signals of the CS and US are repeatedly transferred to the CGC. Then the CGC activates the N3t cell. Our data showed that this excitatory monosynaptic connection from the CGC to the postsynaptic cells (i.e., the B1 motor neuron or the N3t cell) was not changed, but the EPSPs occurred spontaneously in the B3 motor neuron increased in their amplitude in the taste aversion-trained snails. This is the memory trace in the neural circuitry in *Lymnaea* by taste aversion training. The measurement of the size of B3 EPSPs was thought to act as a monitor of the amount of neurotransmitter released from the N3t cell. Because previous studies demonstrated that the B3 motor neuron did not alter the CPG activity directly [22], [35], we do not know the function of this increase in the B3 EPSP amplitude.

The full width at half maximum of the B3 EPSPs was not significantly different among the CNSs isolated from the taste aversion-trained snails, the backward-conditioned snails and the naive control snails. In addition, the N3t cell generates monosynaptically EPSP in the B3 motor neuron [22], [23]. Therefore, we expected that the increase in the B3 EPSP amplitude results from the increase in the amount of neurotransmitter released from the N3t cell but not from the change in the membrane property (i.e., input resistance) of the B3 motor neuron.

The present results are in good agreement with our previous data [9]. In our previous study, we examined a change in the electrical properties of the CGCs after taste aversion training. No significant differences were found between the taste aversion-trained snails and the control snails in terms of the resting potential, the input resistance, the full width at half maximum of the spontaneous action potential, the full width at half maximum of the after-hyperpolarization of spontaneous action potential, or the threshold for an action potential. Further, we examined changes in the N1M cells. No significant differences were found in the resting potential between the taste aversion-trained snails and the control snails. Considering these results together, we thus conclude that the change in the amount of neurotransmitter released form the N3t cell is important for suppression of the N1M cell in the feeding CPG, at least in our target synaptic connections of the CGC-N3t-N1M cells.

Our data showed that the change in the CGC-N3t synaptic connection did not remain as a memory trace, but this result did not rule out the possibility that the CGC-N3t synaptic connection is enhanced during the training or shortly after the training, i.e., during the period of short-term memory. The importance of the cAMP/cAMP response element-binding protein cascade in learning and memory is conserved across phyla. The CGC contains the cAMP response element-binding proteins [29]–[34] and enhances the output response to the following neurons through the assistance of cAMP and the cAMP response element-binding proteins.

### Semi-intact preparation vs. isolated CNS

The importance of the function of N3t cell has also been reported in taste appetitive training in *Lymnaea* [20]. Marra et al. noted that the frequency of tonic N3t firing is reduced following one-trial training using semi-intact preparations [20]. The frequency of the N3t input recorded in the B3 motor neuron is inversely correlated with the strength of the conditioned response, i.e., the conditioned response measured as changes in the fictive feeding response to the CS is stronger in preparations displaying a lower level of inhibition. These previous authors also considered that this is a part of the memory trace for their reward conditioning.

In our present study of taste aversion training, we failed to find an increase of N3t input recorded in the B3 motor neuron. We think that this was due to the difference of preparations, because we used the isolated CNS rather than a semi-intact preparation. For example, when using semi-intact preparations that include the buccal mass and esophagus, the fictive feeding rate increases in response to the application of sucrose to the lips [36]. However, when using semi-intact preparations without the buccal mass and esophagus, the fictive feeding rate does not increase as much following the application of sucrose to the lips [35], [37]. That is, the N3t firing rate in the isolated CNS that does not contain the buccal mass, esophagus or lips may not be suitable for detection of a training effect. The merits of the use of the isolated CNS are as follows: the stable recordings because of the lack of muscle movement and the ease of sample preparations.

### Taste discrimination

Even though the CGC-N3t-N1M synaptic connections, particularly at the N3t cell, is changed after taste aversion training for a specific food taste (i.e., sucrose), we know that snails have the capacity to discriminate between that taste and a different safe taste [24]. That is, taste aversion training does not alter the ability of a safe taste to elicit a feeding response. These results suggest that the loci for taste discrimination occur upstream from the CGC-N3t-N1M synaptic connections. This is a future issue to be studied.

Finally, we note that investigators for CTA in mammals such as rats are often surprised to see that simple invertebrates such as *Lymnaea* are capable of acquiring the conditioned responses by taste aversion training, and that the data using *Lymnaea* give rise to the impression that the neural circuitry required for this learning is fairly primitive [38]. As Bernstein commented, “although this may be true in the case of slugs and pond snails, it is not necessarily the case for mammals such as the rat [38].” We think that this potential weakness is actually an advantage, because the use of simple invertebrate systems can provide answers to important basic questions before moving on to more complex mammalian systems.
